# Deficiency of the RIβ subunit of protein kinase A causes body tremor and impaired fear conditioning memory in rats

**DOI:** 10.1038/s41598-021-81515-x

**Published:** 2021-01-21

**Authors:** Hieu Hoang Trung, Toru Yoshihara, Akito Nakao, Katsumi Hayashida, Yoshiki Hirata, Koumei Shirasuna, Mitsuru Kuwamura, Yuki Nakagawa, Takehito Kaneko, Yasuo Mori, Masahide Asano, Takashi Kuramoto

**Affiliations:** 1 Laboratory of Animal Nutrition, Department of Animal Science, Faculty of Agriculture, Tokyo University of Agriculture, 1737 Funako, Atsugi, Kanagawa 243-0034 Japan; 2 Institute of Laboratory Animals, Graduate School of Medicine, Kyoto University, Kyoto, 606-8501 Japan; 3 Department of Synthetic Chemistry and Biological Chemistry, Graduate School of Engineering, Kyoto University, Kyoto, 615-8510 Japan; 4 Laboratory of Animal Reproduction, Department of Animal Science, Faculty of Agriculture, Tokyo University of Agriculture, 1737 Funako, Atsugi, Kanagawa 243-0034 Japan; 5 Laboratory of Veterinary Pathology, Graduate School of Life and Environmental Science, Osaka Prefecture University, 1-58 Rinkuuourai-kita, Izumisano, Osaka 598-8531 Japan; 6 Department of Chemistry and Biological Sciences, Faculty of Science and Engineering, Iwate University, 4-3-5 Ueda, Morioka, Iwate 020-8551 Japan

**Keywords:** Genetic engineering, Animal breeding, Behavioural genetics, Mutation, Movement disorders, Genetics of the nervous system, Long-term potentiation

## Abstract

The RIβ subunit of cAMP-dependent protein kinase (PKA), encoded by *Prkar1b*, is a neuronal isoform of the type I regulatory subunit of PKA. Mice lacking the RIβ subunit exhibit normal long-term potentiation (LTP) in the Schaffer collateral pathway of the hippocampus and normal behavior in the open-field and fear conditioning tests. Here, we combined genetic, electrophysiological, and behavioral approaches to demonstrate that the RIβ subunit was involved in body tremor, LTP in the Schaffer collateral pathway, and fear conditioning memory in rats. Genetic analysis of WTC-*furue*, a mutant strain with spontaneous tremors, revealed a deletion in the *Prkar1b* gene of the WTC-*furue* genome. *Prkar1b*-deficient rats created by the CRISPR/Cas9 system exhibited body tremor. Hippocampal slices from mutant rats showed deficient LTP in the Schaffer collateral–CA1 synapse. Mutant rats also exhibited decreased freezing time following contextual and cued fear conditioning, as well as increased exploratory behavior in the open field. These findings indicate the roles of the RIβ subunit in tremor pathogenesis and contextual and cued fear memory, and suggest that the hippocampal and amygdala roles of this subunit differ between mice and rats and that rats are therefore beneficial for exploring RIβ function.

## Introduction

Cyclic AMP plays a role as a second messenger and regulates many different cellular functions. Its effects are mediated by cAMP-dependent protein kinase A (PKA). The PKA holoenzyme is a tetramer comprised of two catalytic subunits and a regulatory subunit dimer containing either the regulatory type I (RI) or regulatory type II (RII) subunit^1,2^. The activation of PKA is controlled by the regulatory subunits. Binding of cAMP to the regulatory subunit causes release of the catalytic subunit from the holoenzyme so that it can phosphorylate a broad spectrum of proteins, such as transcription factors, hormone receptors, and ion channels^2,3^. Differences in the anatomical distribution of the regulatory subunits may help identify the diverse effects of cAMP^4^.

Among the regulatory subunits of PKA, the regulatory type I beta (RIβ) subunit, which is encoded by the *Prkar1b* gene in mice, is unique to nervous system tissue^5^ and is thought to play important roles in neuronal functions. The RIβ subunit is involved in hippocampal long-term potentiation (LTP) in the mossy fiber pathway^6^, in long-term depression (LTD) in the Schaffer collateral pathway^7^, and in the processing of nociceptive pain in inflammatory conditions^8^. In addition, the L50R variant of the human *PRKAR1B* gene has been associated with a late-onset neurodegenerative disorder characterized by frontotemporal dementia and parkinsonism^9^. Thus, the RIβ subunit plays critical roles in the nervous system.

Defects of the cAMP/PKA pathway have been found to be involved in various diseases^2^, but little is known about the RIβ subunit. Possible reasons are that *Prkar1b* is expressed only in particular tissues and that *Prkar1b*-deficient mice exhibit no apparent clinical phenotypes^6,7^.

Rat mutants exhibiting spontaneous body tremor that appeared after weaning were discovered in a WTC-*Atrn*^*zi*^ colony in 2000^10^. The tremor phenotype was inherited in an autosomal recessive manner and the causative gene was named “*furue*,” which means “tremor” in Japanese. After removal of the *Atrn*^*zi*^ mutation^11^, a mutant strain, WTC-*furue*, was established.

In the present study, we identified a deletion in the *Prkar1b* gene of the *furue* genome. We produced *Prkar1b*-deficient rats by the CRISPR/Cas9 system and confirmed that *furue* is a loss-of-function mutation of the *Prkar1b* gene. Furthermore, we performed electrophysiological and behavioral studies in *Prkar1b*-deficient rats and showed that they exhibited defects in hippocampal LTP and hippocampus-associated behavior.

## Results

### *Furue* rats harbored a deletion in the *Prkar1b* gene

**Figure 1:**
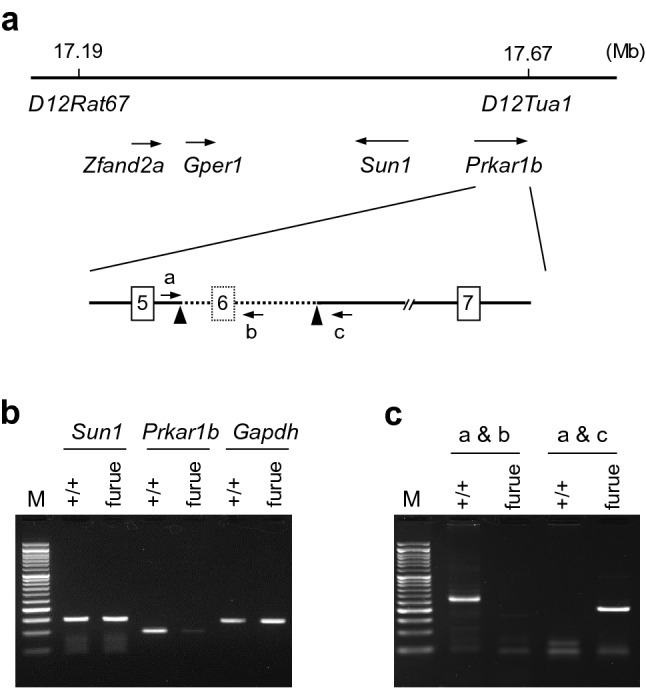
Positional cloning of *furue*. (**a**) Physical map of the *furue* locus. The *furue* locus was defined by *D12Rat67* and *D12Tua1*. Their physical positions are shown above the map (Rnor_6.0 assembly). The positions and directions of four characterized genes are indicated by horizontal arrows below the map. The deletion containing exon 6 (62 bp in length) of the *Prkar1b* gene of the *furue* genome is represented by a dashed line. Breakpoints of the deletion are indicated by arrowheads. Primers to detect the wild-type (a and b) and deletion (a and c) alleles are indicated by arrows. (**b**) Semi-quantitative RT-PCR analysis of *Sun1* and *Prkar1b* mRNA. M, 50-bp DNA ladder molecular marker. (**c**) Genomic DNA PCR for genotyping the *furue* deletion. Primers a, b, and c represent rPrkar1b-wild-F, rPrkar1b-wild-R, and rPrkar1b-del-R, respectively. The positions of primers are depicted in Supplementary Fig. S2.

To identify *furue*, we employed a positional cloning approach. The *furue* locus was mapped to the region that was defined by *D12Rat67* and *D12Tua1* (Supplementary Fig. S1). The physical positions of *D12Rat67* and *D12Tua1* were 17.19 Mb and 17.67 Mb, respectively, in the rat genome assembly Rnor_6.0. Thus, the *furue* locus was estimated to be approximately 480 kb in length (Fig. 1a). Within this region, four characterized genes (zinc finger AN1-type containing 2A (*Zfand2a*); G protein-coupled estrogen receptor 1 (*Gper1*); Sad1 and UNC84 domain containing 1 (*Sun1*); protein kinase, cAMP-dependent regulatory type I beta (*Prkar1b*)) and six uncharacterized genes were mapped. As mice lacking *Sun1* or *Prkar1b* exhibited defects in the CNS^6,12^, we considered *Sun1* and *Prkar1b* to be good candidates and examined their mRNA expression levels in the brain of *furue* rats. Compared to wild-type, the expression level of *Sun1* mRNA in *furue* rats was similar but that of *Prkar1b* mRNA was significantly lower (Fig. 1b, Supplementary Fig. S2). We then sequenced every exon of the *Prkar1b* gene in *furue* rats and found a 5902-bp deletion containing exon 6 (Supplementary Fig. S3), which we confirmed in the *furue* genome (Fig. 1c, Supplementary Fig. S4). As exon 6 was 62 bp in length, the resultant mRNA was deduced to induce a premature stop codon that led to nonsense-mediated decay.

### *Prkar1b*-deficient rats exhibited body tremor

**Figure 2:**
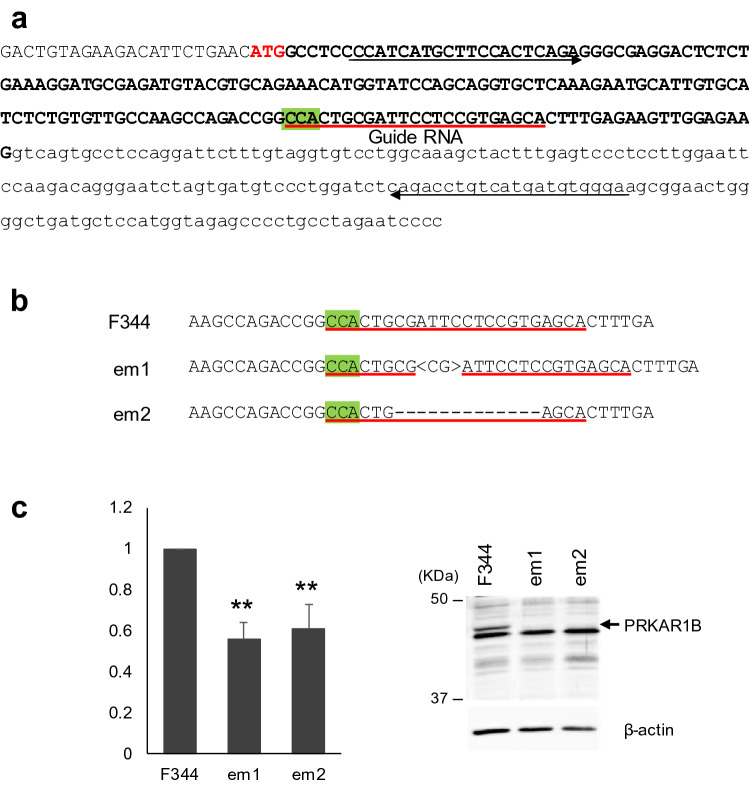
Development of *Prkar1*-deficient rats. (**a**) Target sequence of guide RNA in the CRISPR/Cas system. Exon 2 and intron 2 of the rat *Prkar1b* gene are represented in upper- and lowercase, respectively. The coding sequence is represented in bold. The target sequence of the guide RNA is underlined and the PAM sequence is highlighted in green. Primer sequences to detect mutations induced by the CRISPR/Cas system are indicated by horizontal arrows. (**b**) Genomic alternations induced by the CRISPR/Cas system. A 2-bp insertion was found in the target site of the *Prkar1b*^*em1*^ line. A 13-bp deletion was found in the target site of the *Prkar1b*^*em2*^ line. (**c**) Relative expression level of the *Prkar1b* transcript (left) and expression level of PRKAR1B protein (right) in the hippocampus of *Prkar1b*-deficient rats. Expression levels of *Prkar1b* transcripts are significantly decreased in mutant lines. PRKAR1B proteins are diminished in mutant lines. **P < 0.01.

To examine whether the deletion was causative of *furue*, we produced *Prkar1b*-deficient rats using genome editing. We targeted exon 2 (Fig. 2a), as this exon is thought to be shared among all variants of the *Prakar1b* transcript^13^. Two *Prkar1b*-deficient rat strains were produced. One, F344-*Prkar1b*^*em1*^/Tua, harbored an insertion of 2 bp in the target site. The other, F344-*Prkar1b*^*em2*^/Tua, harbored a deletion of 13 bp in the target site (Fig. 2b). Each mutation was deduced to lead to a frame shift and a premature stop codon in the *Prkar1b* transcripts. *Prkar1b* mRNA was significantly reduced in the hippocampus of these lines: the relative expression levels of *Prkar1b* mRNA compared with those in the wild-type F344 rat were 0.56 ± 0.08 (*P* < 0.01) in F344-*Prkar1b*^*em1*^/Tua and 0.61 ± 0.12 (*P* < 0.01) in F344-*Prkar1b*^*em2*^/Tua (Fig. 2c). Expressions of PRKAR1B protein were diminished in both lines (Fig. 2c, Supplementary Fig. S5). *Prkar1b*-deficient rats exhibited spontaneous tremor after weaning, particularly during walking and rearing (Supplementary Video 1), and the tremor was evident on the lateral sides of the trunk. No sex differences were observed. Tremors appeared 89.2 ± 21.0 times (mean ± SD) during each 60-min observation, with a cumulative duration of 1,487 ± 608.3 s. Thus, the average duration per tremor was 17.6 ± 7.49 s (Table 1). Thus, we concluded that the loss of function of the *Prkar1b* gene was causative of the tremor phenotype in rats.

**Table 1:** Number and duration of tremors during a 60-min observation period in *Prkar1b*-deficient rats.

	Number of tremors	Total duration of tremors (s)	Duration per tremor (s)
Male	98.0 ± 29.4	1311 ± 586.9	15.6 ± 8.40
Female	84.8 ± 13.1	1575 ± 599.7	18.5 ± 6.78
Both	89.2 ± 21.0	1487 ± 608.3	17.6 ± 7.49

Data are represented as mean ± SD.

Data were obtained from 17- to 28-week-old male (n = 3) and 19- to 28-week-old female (n = 6) rats. There was no difference between sexes in the number of tremors (*P* = 0.30), total tremor duration (*P* = 0.31), or duration per tremor (*P* = 0.35).

### *Prkar1b*-deficient rats exhibited abnormal LTP

**Figure 3:**
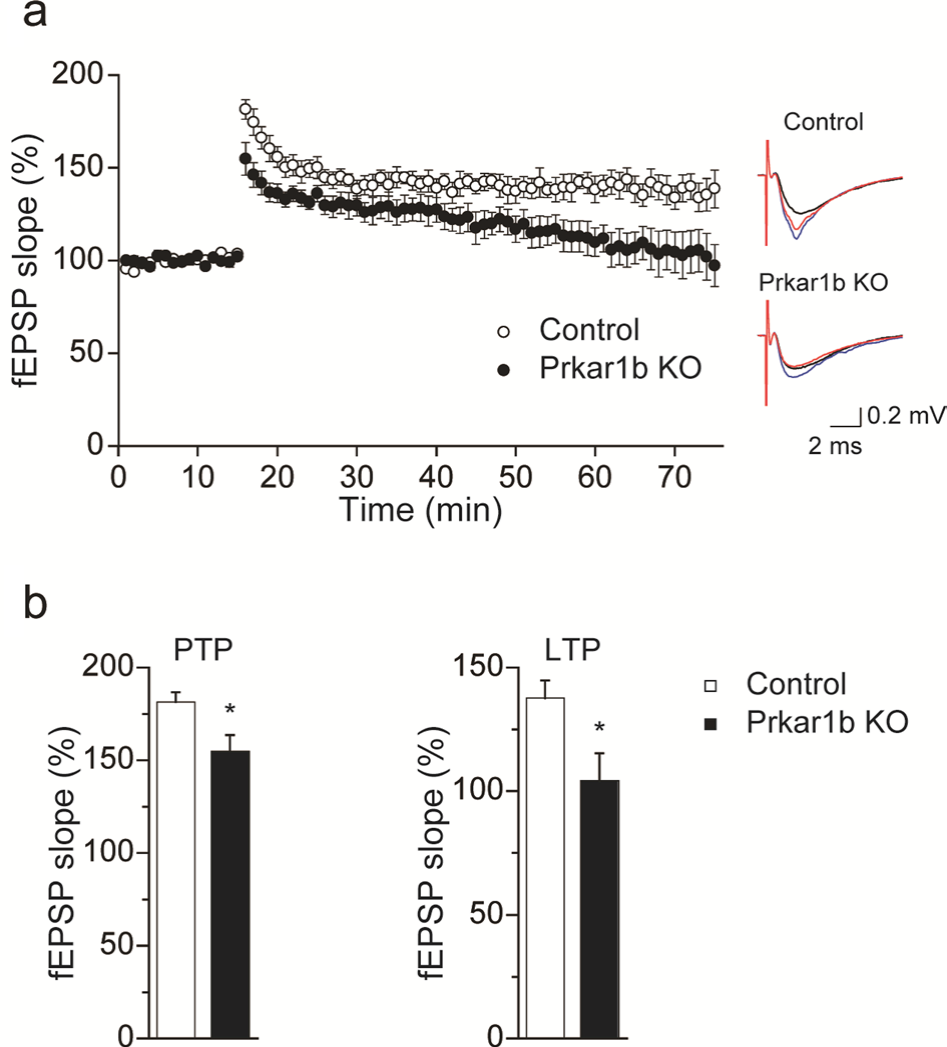
Impaired hippocampal synaptic plasticity at the Schaffer collateral–CAl synapse in *Prkar1b*-deficient rats. (**a**) Field potentials were recorded from the CA1 region of hippocampal slices from wild-type and *Prkar1b*-deficient rats. LTP was induced by theta burst conditioning stimulation (TBS). The slope of the EPSP relative to the pre-TBS level is plotted (wild-type, n = 6 slices from four rats; *Prkar1b*-deficient, n = 6 slices from three rats). Data are expressed as mean ± SEM. The inset traces show fEPSP at baseline (black), PTP (blue), and LTP (red) in wild-type (upper) and *Prkar1b*-deficient (lower) rats. (**b**) Changes of fEPSP amplitude compared with baseline. Regarding PTP at 1 min after TBS, the change observed in *Prkar1b*-deficient rats was significantly smaller than that in wild-type rats. Regarding LTP during 50–60 min after TBS, the change observed in *Prkar1b*-deficient rats was significantly smaller than that in wild-type rats. Data are expressed as mean + SEM. *P < 0.05.

PRKAR1B was involved in LTP in the mouse hippocampus^6^. To clarify the effect of *Prkar1b* deficiency on rat hippocampal synaptic plasticity, we examined LTP in the hippocampus of *Prkar1b*-deficient rats. These rats exhibited reduced LTP induction and maintenance at the Schaffer collateral–CAl synapse following theta burst stimulation (Fig. 3a). Regarding post-tetanic potentiation (PTP), at 1 min after LTP induction the slope of the field excitatory postsynaptic potential (fEPSP) was augmented by 181.4 ± 5.3% in slices from wild-type but only by 154.8 ± 8.7% in slices from *Prkar1b*-deficient rats (t = − 2.61, *P* = 0.026) (Fig. 3b). Regarding LTP, the fEPSP slope of *Prkar1b*-deficient rats returned to the baseline level 50–60 min after theta burst stimulation, while the fEPSP slope in wild-type slices was consistently increased by 137.6 ± 7.2% (t = − 2.50, *P* = 0.031) (Fig. 3b). These findings indicated that the induction and maintenance of LTP at the Schaffer collateral–CA1 synapse were defective in the hippocampal slices from *Prkar1b*-deficient rats.

### *Prkar1b*-deficient rats exhibited abnormal nociception-, hippocampus- and amygdala-related behaviors

**Figure 4:**
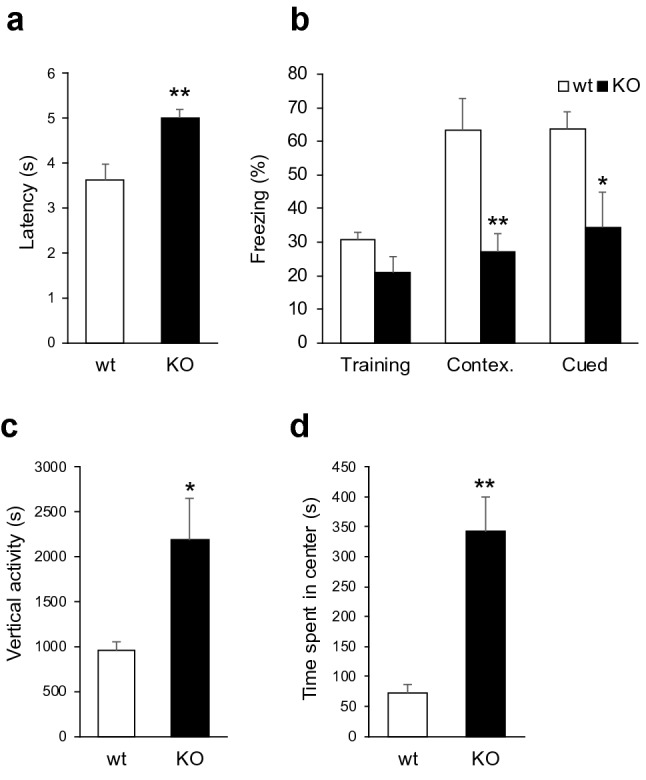
Hot plate, fear conditioning, and open-field tests. (**a**) The time spent by rats on the hot plate. The latency to lick a hind paw, stomp, or jump from the hot plate was significantly increased in *Prkar1b*-deficient rats. (**b**) The duration of freezing (%) in the fear conditioning tests. The freezing of *Prkar1b*-deficient rats was significantly decreased compared with that of wild-type rats in the contextual and cued trials, but not in the training trial. (**c**) The duration of vertical activity in the open-field test was significantly increased in *Prkar1b*-deficient rats compared with wild-type rats. (**d**) *Prkar1b*-deficient rats stayed in the central area of the open field significantly longer than wild-type rates. Data are expressed as mean + SEM. *P < 0.05, **P < 0.01.

*Prkar1b* is known to be involved in the processing of nociceptive pain in inflammatory conditions^8^. To examine the nociception of *Prkar1b*-deficient rats, we performed the hot plate test. The latency to lick a hind paw, stomp, or jump from the hot plate was significantly longer in *Prkar1b*-deficient rats than in wild-type rats (t = − 2.97, *P* = 0.0071) (Fig. 4a). This indicated that pain sensitivity was decreased in the mutant rats.

Given that *Prkar1b*-deficient rats lack CA1 LTP, we explored their learning capabilities. The hippocampus is thought to construct an internal representation of the spatial properties of an environment^14^. Lesions of the hippocampus produce several defects in exploration-related behavior, including increased activity, reduced spontaneous alternation behavior, and decreased habituation to sensory stimulation^15^. Thus, we explored contextual conditioning to fear and the response to novelty in *Prkar1b*-deficient rats. In the fear conditioning test, animals learn to fear a new environment (context) or an emotionally neutral conditioned stimulus, such as light or tone, because of its temporal association with an aversive unconditioned stimulus, usually foot shock. When exposed to the same context or the same cue, conditioned animals show freezing behavior^16,17^.

In the training trial, the freezing duration of *Prkar1b*-deficient rats did not differ from that of wild-type rats. In contrast, the freezing duration of *Prkar1b*-deficient rats was significantly shorter than that of wild-type rats in both the contextual trial (t = − 2.78, *P* = 0.0097) and the cued trial (t = − 2.24, *P* = 0.024) (Fig. 4b).

In the open-field test, there was no significant difference in the duration of spontaneous activity between wild-type and *Prkar1b*-deficient rats (data not shown). The duration of vertical activity was significantly increased in *Prkar1b*-deficient rats compared to wild-type rats (t = 2.51, *P* = 0.015) (Fig. 4c). In addition, the time spent in the central part of the open field was significantly higher in *Prkar1b*-deficient rats (t = − 4.66, *P* = 0.0004) (Fig. 4d).

## Discussion

In the present study, we revealed that deficiency of the *Prkar1b* gene was causative of tremor in rats. The *Prkar1b* gene encodes the RIβ subunit of PKA; this subunit is known to be expressed dominantly in the nervous system and to regulate the activity of PKA^18^. *Prkar1b* is involved in the modulation of hippocampal neural plasticity^6,7^ and nociceptive processing^8^, and shows an association with neurodegenerative disorders^9^. However, *Prkar1b*-deficient mice exhibit no body tremor^6^. Thus, our present study demonstrated for the first time that *Prka1b* is involved in the expression of tremor.

The tremor of the *Prkar1b*-deficient rats was similar to that of *furue* rats even though the two types of rats exhibit disruption of different exons. In the *Prkar1b*-deficient rats, PRKAR1B protein levels were diminished (Fig. 2c). Although we did not examine the expression levels of PRKAR1B in *furue* rats, it is very likely that these would also be diminished, because the causative deletion in the *furue* genome is believed to induce a premature stop codon that leads to nonsense-mediated decay. Thus, we consider that the PRKAR1B protein is reduced in both mutants, thus causing the tremor phenotype.

Body tremors may originate centrally and/or peripherally. Although the neural basis of the tremor in *Prkar1b* mutants was not examined in the present study, the fact that the *Prkar1b* transcripts were specifically expressed in the brain but not the skeletal muscle^19^ suggests that the tremors of the *Prkar1b* mutants may be centrally mediated.

Congenital body tremor is known to result from the deficiency of various genes, such as those for ion channels, neurotransmitter receptors, and myelin proteins. Given that PRKAR1B can regulate PKA activity in neuronal cells, *Prkar1b* deficiency can lead to the dysregulation of these channels and receptors through their phosphorylation. For example, mutations in the Shaker-like voltage-gated potassium channel Kv1.1 (KCNA1) are known to cause tremor and temporal lobe epilepsy^20,21^. The KCNA1 channel is phosphorylated by PKA and thereby induces a change in membrane potential^22^. In addition, mutation of the GABA_A_ receptor gene causes intention tremor in mice^23^, and the GABA_A_ receptor is phosphorylated by PKA^24^. Although further investigations are required, we hypothesize that defects in PKA-induced phosphorylation of tremor-associated ion channels and/or receptors are one mechanism underlying the expression of tremor in WTC-*furue* and *Prkar1b*-deficient rats.

In this study, hippocampal slices from *Prkar1b*-deficient rats demonstrated a defect of LTP at the Schaffer collateral–CA1 synapse (Fig. 3), which is consistent with a previous finding that the activity of PKA is critical for the generation of LTP in area CA1 of the rat hippocampus^25,26^. By contrast, mice deficient for *Prkar1b* exhibited normal LTP at the Schaffer collateral–CA1 synapse^7^. Thus, our finding suggests that PRKAR1B may play a different role in the generation of LTP at the Schaffer collateral–CA1 synapse in rats and mice. We hypothesize that this phenotypic discrepancy may result from species-specific differences in expression levels of molecules involved in LTP at the Schaffer collateral–CA1 synapse, or the ability of these molecules to compensate for PRKAR1B deficiency.

The duration of freezing behavior of the conditioned *Prkar1b*-deficient rats decreased significantly in the contextual trial (Fig. 4b). This is likely to be due to defects in LTP in the Schaffer collateral pathway in these rats, because this LTP plays an important role in spatial learning and contextual conditioning^27^. *Prkar1b*-deficient rats also exhibited a decreased duration of freezing behavior in the cued trial (Fig. 4b). This strongly suggested hypofunction of the amygdala, because the amygdala is one of the control centers for emotional states such as fear and anxiety^28^ and it plays an important role in cued fear conditioning^29^. In addition, the reduced pain sensitivity could confer the observed reduced fear memories, due to interference with the fear memory acquisition.

In the open-field test, *Prkar1b*-deficient rats exhibited increased durations of vertical activity and time spent in the center of the arena (Fig. 4c,d), suggesting that they required more time to acquire spatial knowledge and/or felt less fear or anxiety. The hippocampus and the amygdala have been implicated in spatial cognition^30,31^ and emotional states^28^, respectively. Our fear conditioning test demonstrated that *Prkar1b*-deficient rats had a defect in hippocampus-related memory and hypofunction of the amygdala. Thus, we theorized that these rats’ increased activity and greater time spent in the center might result from either impaired navigation ability, potentially due to a hippocampal deficit, and/or insufficient fear or anxiety, possibly due to a defect in the amygdala.

We demonstrated that *Prkar1b*-deficient rats exhibited tremor and abnormal behaviors associated with hippocampal functions. By contrast, *Prkar1b*-deficient mice are not known to exhibit such behaviors^6,7^. Phenotypic discrepancies have been demonstrated between mice and rats in which the orthologous gene was disrupted. For example, while ataxia telangiectasia mutated (*Atm*) deficiency does not cause neurodegeneration in mice, it does in rats, which develop hind limb paralysis like human patients^32^. Severe combined immunodeficiency (SCID) rats, which lack the protein kinase, DNA activated, catalytic polypeptide gene (*Prkdc*), show several phenotypic differences from SCID mice, including growth retardation, premature senescence, and more severe immunodeficiency without "leaky" phenotypes^33^. Thus, our present study highlights the importance of cross-species comparisons when researching both novel and established gene functions, and suggests an advantage of *Prkar1b*-deficient rats for exploring the role of PRKAR1B in cellular mechanisms that underlie neurological disorders, learning, and memory.

In summary, *Prkar1b* gene deficiency caused body tremor as well as defects in LTP in the Schaffer collateral pathway and in hippocampus-related memory. These findings strongly suggest a role of the RIβ subunit of PKA in tremor expression and in LTP generation in the hippocampus in rats.

## Methods

### Ethical use of animals

All animal experiments were approved by the Animal Research Committees of Kyoto University and Tokyo University of Agriculture and were conducted according to their regulations on animal experimentation. This study was carried out in compliance with the ARRIVE guidelines.

### Animals

ACI/NJcl and BN/SsNSlc rats were purchased from CLEA Japan, Inc. (Meguro, Tokyo, Japan) and Japan SLC, Inc. (Hamamatsu, Shizuoka, Japan), respectively. F344/Stm rats were supplied by the National BioResource Project-Rat (NBRP-Rat) in Japan^34^.

### Positional cloning

To localize the *furue* locus to a specific chromosomal region, we produced a total of 67 (ACI/NJcl × WTC-*furue*)F1 × WTC-*furue* backcross progeny and performed genome-wide scanning of DNA samples using a panel of 53 simple sequence length polymorphism (SSLP) markers that covered all autosomal chromosomes. To narrow down the *furue* locus, we produced a total of 300 (BN/SsNSlc × WTC-*furue*)F1 × WTC-*furue* backcross progeny and genotyped DNA samples using 24 existing and one newly-developed (*D12Tua1*; 5′-CGCTTAACCTCCCGTGTCT-3′ and 5′-TGTGGAAATGTGAGGACTCG-3′) SSLP markers on chromosome 12. To identify mutations, every exon of the *Prkar1b* gene of WTC-*furue* rats was sequenced. Genotyping of the *furue* deletion was performed by PCR using the following primers: rPrkar1b-wild-F, 5′-CTGAGACGGGTGGATTGTCT-3′; rPrkar1b-wild-R, 5′-GGCCTCAAACTCAGGAAAGTT-3′; and rPrkar1b-del-R, 5′-CACCCCCAAAACAAGAAGTC-3′.

### Quantitative PCR

Total RNA was isolated using a FastGene RNA Premium Kit (NIPPON Genetics Co., Ltd., Bunkyo, Tokyo, Japan) according to the manufacturer’s instructions. cDNA was synthetized using FastGene Scriptase II (NIPPON Genetics Co., Ltd.). Real-time PCR was performed using the Thermal Cycler Dice Real Time System (Takara Bio, Inc., Otsu, Shiga, Japan) with a KAPA SYBR Fast qPCR Kit (Roche Diagnostics K.K., Minato, Tokyo, Japan). Primer sequences for quantification of *Prkar1b* cDNA were 5′-CTTCAGTCCTCCAGCGACGAT-3′ and 5′-GGTTCAGCAGCAGGGCAAT-3′, and those for *Sun1* cDNA were 5′-GAGTCAGGCAGGGATTTCAG-3′ and 5′-AGAGCACCGAGTGCTTAGGA-3′. The number of target molecules in a test sample was analyzed by monitoring the amplification curves for both test and reference samples, all of which contained 10^1^ to 10^6^ molecules of the gene of interest. The number of target molecules was normalized to that of peptidylprolyl isomerase A (*Ppia*) as an internal control^35^.

### Western blotting

Western blotting was performed as described previously^36^. Briefly, hippocampus and cerebral cortex tissues were homogenized in an ice-chilled RIPA Buffer (FUJIFILM Wako Pure Chemical Corporation, Chuo, Osaka, Japan) containing protease inhibitors (cOmplete, Sigma-Aldrich Japan K.K., Meguro, Tokyo, Japan). The lysate was electrophoresed on a 10% Bis–Tris gel in 1× MOPS buffer. After transfer onto polyvinylidene fluoride membranes, nonspecific antibody binding was blocked for 1 h at room temperature using ImmunoBlock (DS Pharma Biomedical Co., Ltd., Suita, Osaka, Japan). Membranes were incubated for 24 h at 4 °C with primary antibody in Can Get Signal Immunoreaction Enhancer Solution (Toyobo Co., LTD., Kita, Osaka, Japan). Anti-PRKAR1B antibody (PA5-87652, Thermo Fisher Scientific K.K., Minato, Tokyo, Japan) was used at a dilution of 1:2000, and anti-actin antibody (A3853; Sigma-Aldrich, St. Louis, MO, USA) was used at 1:10,000. The secondary antibody was an anti-rabbit IgG horseradish peroxidase (GE Healthcare, Buckinghamshire, UK) and was used at 1:100,000 in Can Get Signal Immunoreaction Enhancer Solution. Signals were developed by ECL Western Blotting Detection Reagents (GE Healthcare) and detected using an ImageQuant LAS 4000 Image Analyzer (GE Healthcare Japan Corporation, Hino, Tokyo, Japan).

### Development of genetically modified rats by genome editing

Genome editing by CRISPR/Cas was performed as described previously^37^. Briefly, pronuclear-stage embryos of F344/Stm rats were produced by natural mating. The oviducts of female rats with vaginal plugs were removed after euthanasia by CO_2_ and cervical dislocation, and embryos were flushed out from the ampullae with culture medium. Cas9 protein (Integrated DNA Technologies, Inc., Coralville, Iowa, USA) and gRNA were introduced into the embryos using a super electroporator NEPA 21 (NEPA GENE Co., Ltd., Ichikawa, Chiba, Japan). Embryos that developed to the two-cell stage were transferred into the oviducts of pseudopregnant females that were anesthetized using isoflurane. Offspring were genotyped by the Amp-FTA method^38^. Genomic DNA was extracted from blood that adhered to FTA cards. PCR templates were prepared by punching out discs from the FTA cards and placing the discs in Ampdirect Plus buffer (Shimadzu Corporation, Nakagyo, Kyoto, Japan).

### Electrophysiological experiments with a multi-electrode array

Five- to 8-week-old *Prkar1b*-deficient (F344-*Prkar1b*^*em2*^/Tua) (n = 3) and wild-type (n = 4) rats were killed by decapitation after anesthesia with isoflurane. The brain was immediately soaked for approximately 2 min in an ice-cold, oxygenated preparation of artificial cerebrospinal fluid (aCSF) composed of the following (in mM): 124 NaCl, 26 NaHCO_3_, 10 glucose, 3 KCl, 1.25 KH_2_PO_4_, 2 CaCl_2_, and 1 MgSO_4_. Appropriate portions of the brain were trimmed and placed on the ice-cold stage of a vibrating tissue slicer (LinearSlicer PRO7, DOSAKA EM CO., LTD. Sakyo, Kyoto, Japan). The stage was immediately filled with oxygenated and frozen aCSF. The thickness of each tissue slice was 300 µm. Sections were soaked in the oxygenated preparation buffer for 1 h at 27.5 °C. Procedures for electrophysiological experiments with microelectrode array (MED Probe, Alpha MED Scientific, Inc., Ibaraki, Osaka, Japan) were described in previous reports^39^. The device has an array of 64 planar microelectrodes (50 × 50 µm) arranged in an 8 × 8 pattern with interelectrode spacing of 150 µm (MED-P515A, Alpha MED Scientific, Inc.). The slices were positioned to cover the 8 × 8 array on the MED Probe and placed in a small incubator at 32 °C, and responses were collected in aCSF. Fresh, oxygenated aCSF was infused at 1.5 mL/min. Field potentials at all 64 sites were simultaneously recorded with the MED64-Basic System (Alpha MED Scientific, Inc.) at a 20-kHz sampling rate. One of the electrodes in the Schaffer collateral fibers projecting to the CA1 region was selected as the stimulating electrode, while an electrode in the stratum radiatum was selected as the recording electrode. Bipolar constant current pulses were delivered at 30% of the intensity that produced the maximum field excitatory postsynaptic potential (fEPSP). Baseline fEPSPs were recorded for more than 15 min before the conditioning stimulation. LTP was induced by theta burst conditioning stimulation^40^. Theta burst stimulation consists of 10 bursts every 200 ms; each burst comprised four pulses (0.2 ms width) every 10 ms. The fEPSP slopes were expressed as a percentage of the average value measured during the 15-min baseline recording period.

### Behavior analyses

Tremors of *Prkar1b*-deficient rats (F344-*Prkar1b*^*em2*^/Tua) were quantified using 17- to 28-week-old male (n = 3) and female (n = 6) mutant rats. Each rat was placed a box (W 255 mm, D 415 mm, H 195 mm) and allowed to habituate for 15 min, and the number and duration of tremors were then assessed for 60 min.

The fear conditioning, open field, and hot plate tests were performed using 10-month-old *Prkar1b*-deficient (F344-*Prkar1b*^*em2*^/Tua) (n = 6) and wild-type (n = 7) rats. The fear conditioning test was performed over 3 days. The test consisted of three different training and test phases. On the first day, conditioning was conducted in a box (W 327 mm, D 250 mm, H 284 mm, 100 lx) in which the rat was allowed to move freely for 8 min. For the conditioning, white noise (55 dB) and electrical foot shock (0.3 mA, 2 s) were presented simultaneously three times, from 120–150 s, 240–270 s, and 360–390 s. On the second day, freezing performance was measured for 5 min in the same box, this time without white noise or foot shock, to assess contextual fear memory. On the third day, the rat was placed in a different box, this one triangular in shape (W 350 mm, D 350 mm, H 390 mm, 10 lx), to measure cued fear memory. Freezing performance was monitored for 6 min and the same white noise used in the conditioning phase was presented from 180 to 210 s. All of the equipment and the analysis software were purchased from O’HARA Co., Ltd., Tokyo, Japan.

The open-field test was performed in a test chamber (W 400 mm, D 400 mm, H 300 mm) and the animal activity was detected by a photobeam emitter mounted on one wall. The rat was positioned in the left corner of the arena at the beginning of experiment and was permitted to freely explore the arena for 30 min. The total distance moved and the time spent in the central area (encompassing the innermost 30% of the arena area) was automatically calculated. All of the equipment and the analysis software was purchased from Accuscan Instruments, Inc, (Columbus, Ohio, USA).

The hot plate test was performed by placing rats on a hot plate at 55 °C. The durations before paw licking, stomping, and/or jumping from the hot plate were recorded.

### Statistical analysis

Data are expressed as the mean ± SEM. Statistical significance was determined using the two-tailed and unpaired Student’s t-tests. A *P* value of less than 0.05 was considered statistically significant.

## Supplementary Information

Supplementary Legend.

Supplementary Video S1.

Supplementary Figures.
