# Supplementary material for: Deficiency of the RIβ subunit of protein kinase A causes body tremor and impaired fear conditioning memory in rats
Source: Sci Rep. 2021 Jan 21;11:2039. doi: 10.1038/s41598-021-81515-x (PMC7820254; doi:10.1038/s41598-021-81515-x)
Supplement: Supplementary file 1 — Supplementary Legend. [file 41598_2021_81515_MOESM1_ESM.docx]

## Supplementary Figure S1 Genetic mapping of *furue*

a, Fine linkage map around the *furue* locus. This map was made using 300 (BN/SsNSlc × WTC-*furue*)F1 × WTC-*furue* backcross progeny.

b, Haplotypes of backcross progeny carrying recombinant chromosomes between *D12Rat118* and *D12Rat73*. Filled boxes represent rats heterozygous for genetic markers, while open boxes represent rats homozygous for genetic markers. Numbers of the progeny for each haplotype are shown below the haplotype. The *furue* locus was mapped between *D12Rat67* and *D12Tua1*.

## Supplementary Figure S2 Raw image for Fig. 1b

Lanes marked X have been cropped from the gel image shown in Fig. 1b. M; 50-bp DNA ladder molecular marker.

## Supplementary Figure S3 Flanking sequence on either end of the deletion

Arrowheads represent the 5′- and 3′-ends of the deletion (indicated in red) found in the *furue* genome. Horizontal arrows represent primers to detect the wild-type (a & b) and deletion (a & c) alleles, as shown in Figure 1a.

## Supplementary Figure S4 Raw image for Fig. 1c

Lane marked X has been cropped from the gel image shown in Fig. 1c. M; 50-bp DNA ladder molecular marker.

## Supplementary Figure S5 Raw images for Fig. 2c

Multiple exposure images of Western blots with PRKAR1B (left) and Beta-actin (right) antibodies. Exposure time was indicated the left side of each image. The bottom images were used in Fig. 2c. Lanes marked X have been cropped from the blot shown in Fig. 2c. The filter on which proteins were blotted was trimmed according to molecular standards (from ~37 kDa to ~50 kDa) to use the minimum possible amount of hybridization buffer. M; Precision Plus Protein Kaleidoscope Standards (Bio-Rad Laboratories, Inc., Hercules, California, USA).

## Supplementary Video S1 Spontaneous tremor in the *Prkar1b*-deficient rat

A *Prkar1b*-deficient rat, whose tail is colored black, exhibits spontaneous body tremor, but its wild-type littermate (no tail marking) does not.
