# Supplementary material for: Deficiency of the RIβ subunit of protein kinase A causes body tremor and impaired fear conditioning memory in rats
Source: Sci Rep. 2021 Jan 21;11:2039. doi: 10.1038/s41598-021-81515-x (PMC7820254; doi:10.1038/s41598-021-81515-x)
Supplement: Supplementary file 3 — Supplementary Figures. [file 41598_2021_81515_MOESM3_ESM.pptx]

## Slide 1
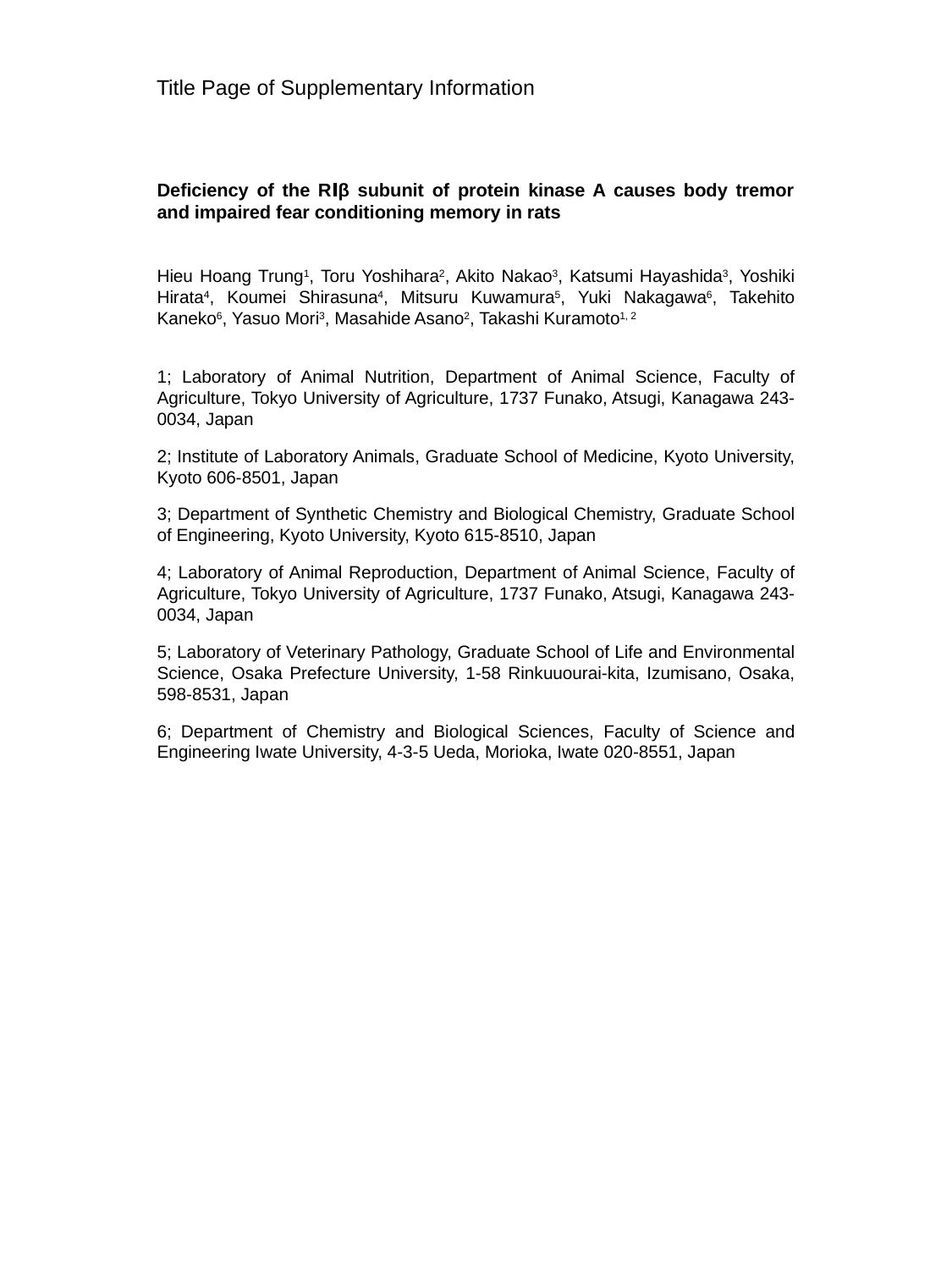

Title Page of Supplementary Information
Deficiency of the RⅠβ subunit of protein kinase A causes body tremor and impaired fear conditioning memory in rats
Hieu Hoang Trung1, Toru Yoshihara2, Akito Nakao3, Katsumi Hayashida3, Yoshiki Hirata4, Koumei Shirasuna4, Mitsuru Kuwamura5, Yuki Nakagawa6, Takehito Kaneko6, Yasuo Mori3, Masahide Asano2, Takashi Kuramoto1, 2
1; Laboratory of Animal Nutrition, Department of Animal Science, Faculty of Agriculture, Tokyo University of Agriculture, 1737 Funako, Atsugi, Kanagawa 243-0034, Japan
2; Institute of Laboratory Animals, Graduate School of Medicine, Kyoto University, Kyoto 606-8501, Japan
3; Department of Synthetic Chemistry and Biological Chemistry, Graduate School of Engineering, Kyoto University, Kyoto 615-8510, Japan
4; Laboratory of Animal Reproduction, Department of Animal Science, Faculty of Agriculture, Tokyo University of Agriculture, 1737 Funako, Atsugi, Kanagawa 243-0034, Japan
5; Laboratory of Veterinary Pathology, Graduate School of Life and Environmental Science, Osaka Prefecture University, 1-58 Rinkuuourai-kita, Izumisano, Osaka, 598-8531, Japan
6; Department of Chemistry and Biological Sciences, Faculty of Science and Engineering Iwate University, 4-3-5 Ueda, Morioka, Iwate 020-8551, Japan

## Slide 2
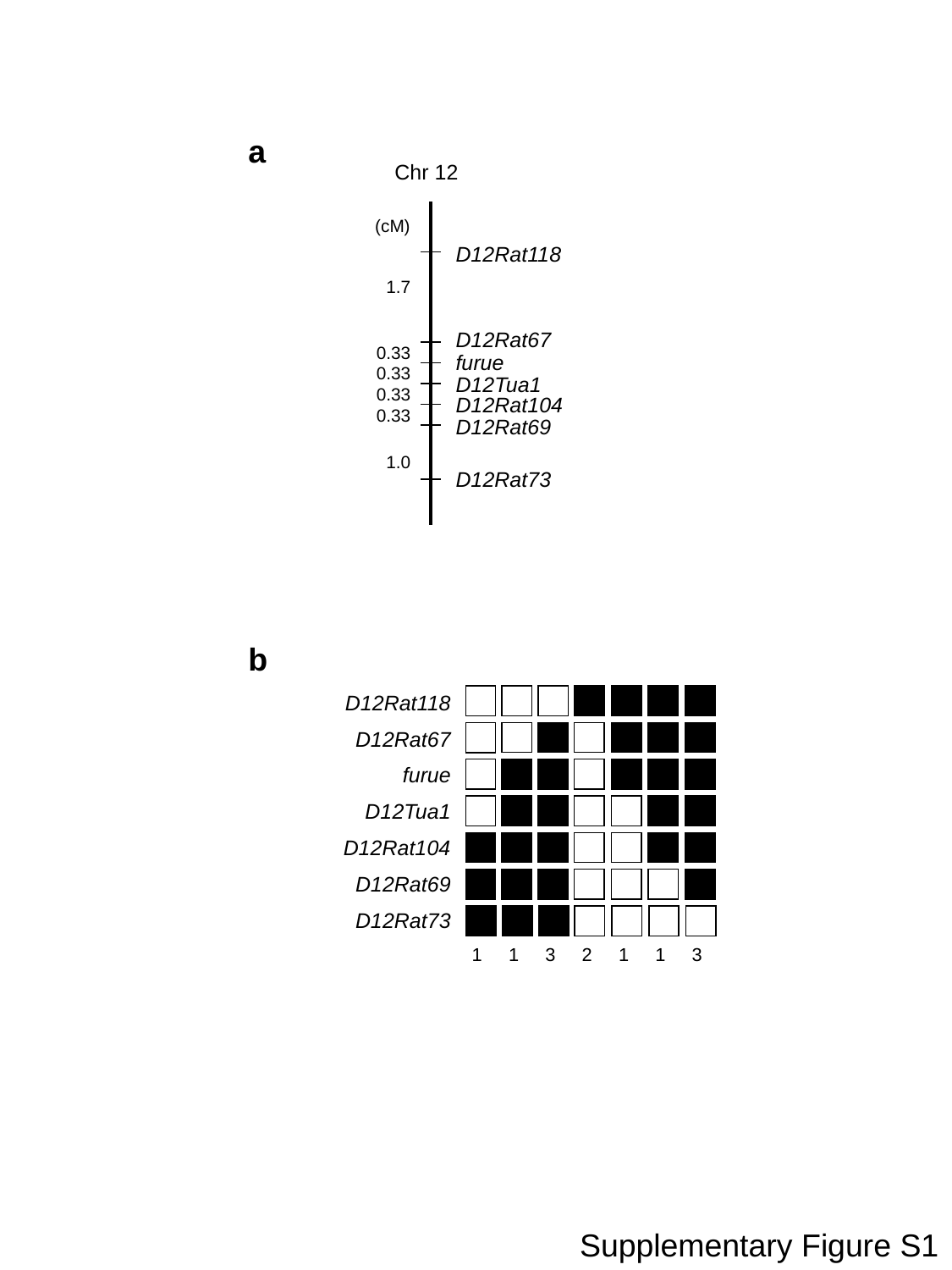

a
Chr 12
(cM)
D12Rat118
1.7
D12Rat67
0.33
furue
0.33
D12Tua1
0.33
D12Rat104
0.33
D12Rat69
1.0
D12Rat73
b
D12Rat118
D12Rat67
furue
D12Tua1
D12Rat104
D12Rat69
D12Rat73
1
1
3
2
1
1
3
Supplementary Figure S1

## Slide 3
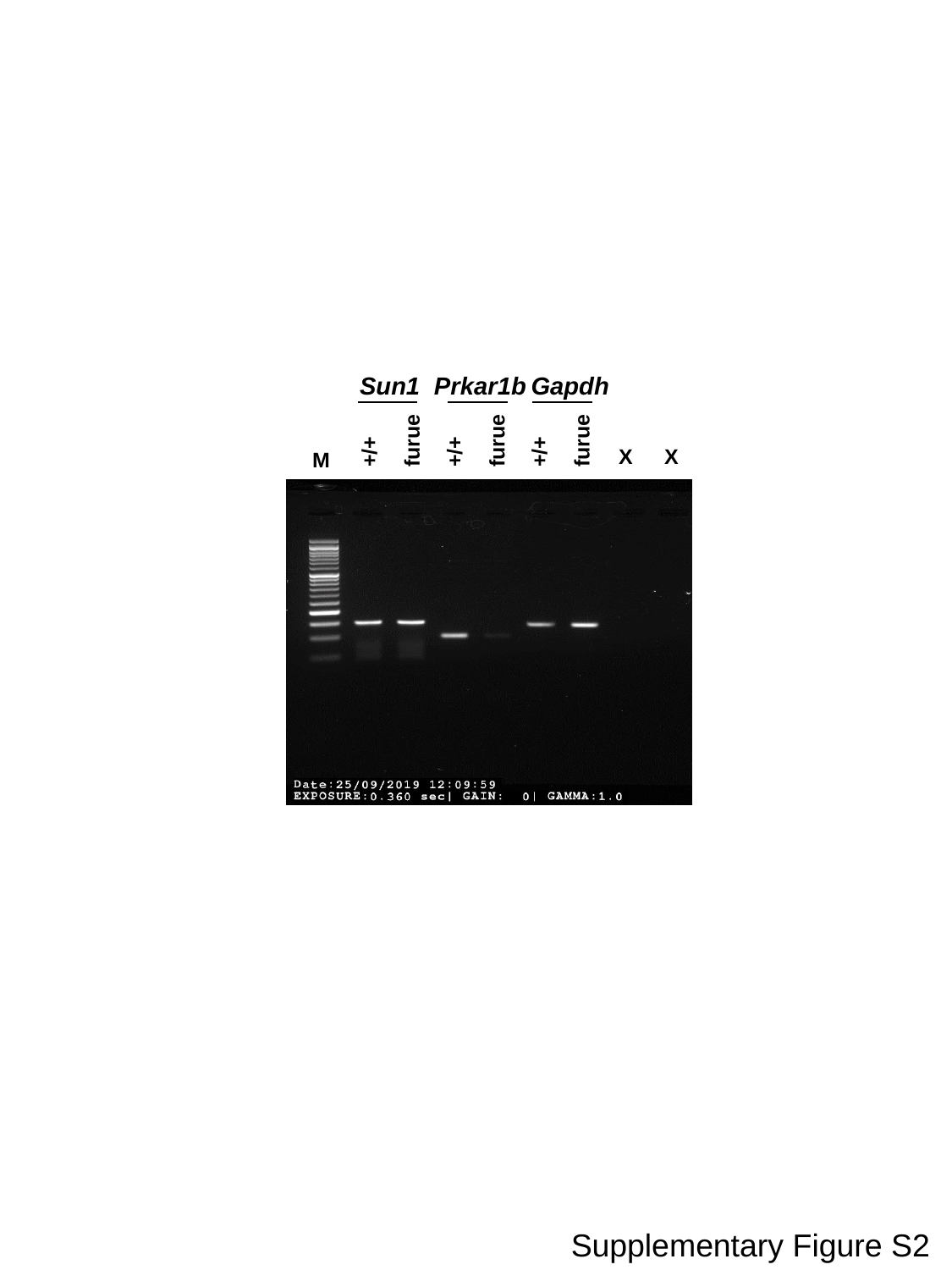

Sun1
Prkar1b
Gapdh
furue
furue
furue
+/+
+/+
+/+
X
X
M
Supplementary Figure S2

## Slide 4
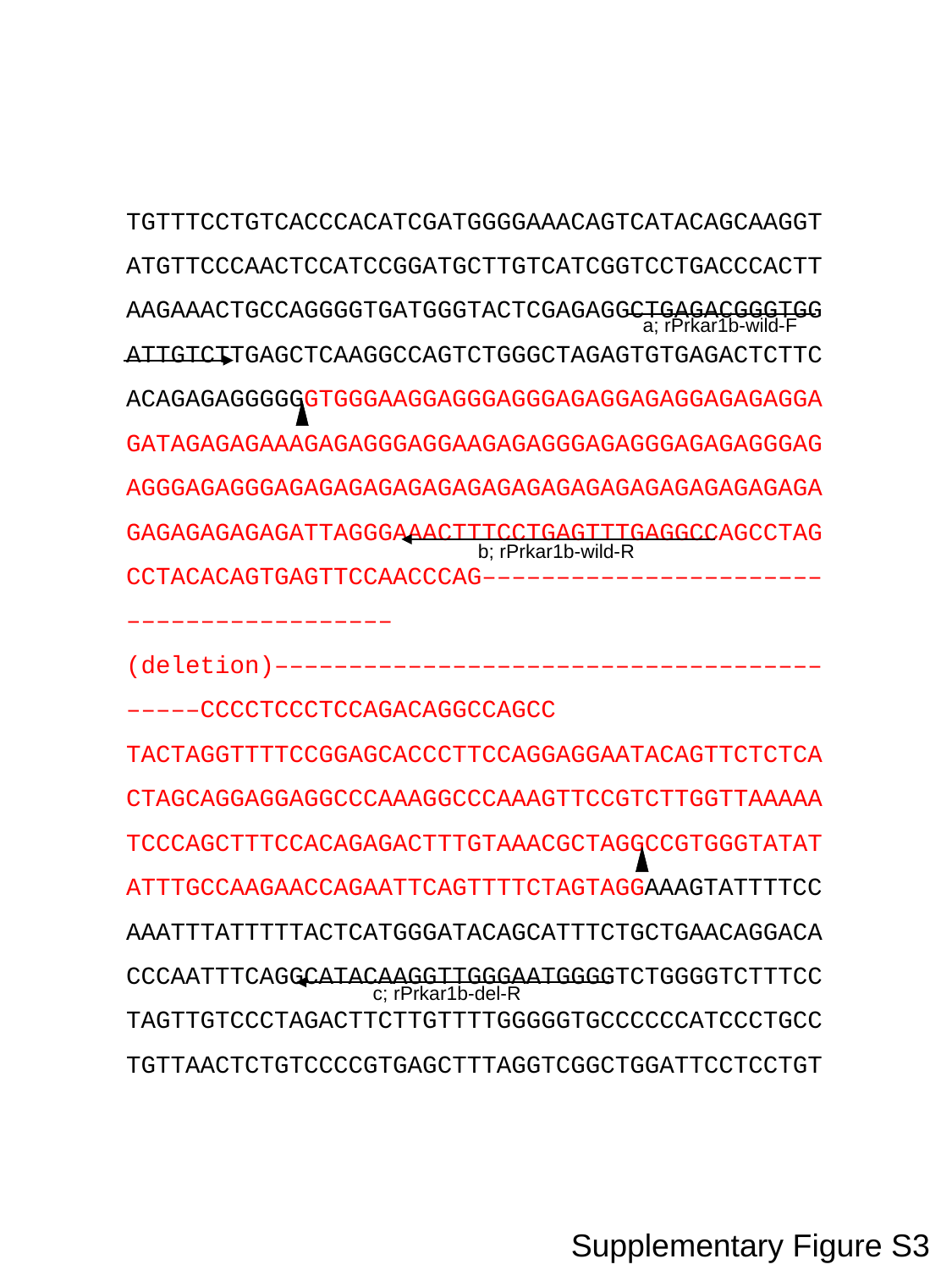

TGTTTCCTGTCACCCACATCGATGGGGAAACAGTCATACAGCAAGGTATGTTCCCAACTCCATCCGGATGCTTGTCATCGGTCCTGACCCACTTAAGAAACTGCCAGGGGTGATGGGTACTCGAGAGGCTGAGACGGGTGGATTGTCTTGAGCTCAAGGCCAGTCTGGGCTAGAGTGTGAGACTCTTCACAGAGAGGGGGGTGGGAAGGAGGGAGGGAGAGGAGAGGAGAGAGGAGATAGAGAGAAAGAGAGGGAGGAAGAGAGGGAGAGGGAGAGAGGGAGAGGGAGAGGGAGAGAGAGAGAGAGAGAGAGAGAGAGAGAGAGAGAGAGAGAGAGAGAGATTAGGGAAACTTTCCTGAGTTTGAGGCCAGCCTAGCCTACACAGTGAGTTCCAACCCAG–––––––––––––––––––––––––––––––––––––––––(deletion)––––––––––––––––––––––––––––––––––––––––––CCCCTCCCTCCAGACAGGCCAGCC
TACTAGGTTTTCCGGAGCACCCTTCCAGGAGGAATACAGTTCTCTCACTAGCAGGAGGAGGCCCAAAGGCCCAAAGTTCCGTCTTGGTTAAAAATCCCAGCTTTCCACAGAGACTTTGTAAACGCTAGGCCGTGGGTATATATTTGCCAAGAACCAGAATTCAGTTTTCTAGTAGGAAAGTATTTTCCAAATTTATTTTTACTCATGGGATACAGCATTTCTGCTGAACAGGACACCCAATTTCAGGCATACAAGGTTGGGAATGGGGTCTGGGGTCTTTCCTAGTTGTCCCTAGACTTCTTGTTTTGGGGGTGCCCCCCATCCCTGCCTGTTAACTCTGTCCCCGTGAGCTTTAGGTCGGCTGGATTCCTCCTGT
a; rPrkar1b-wild-F
b; rPrkar1b-wild-R
c; rPrkar1b-del-R
Supplementary Figure S3

## Slide 5
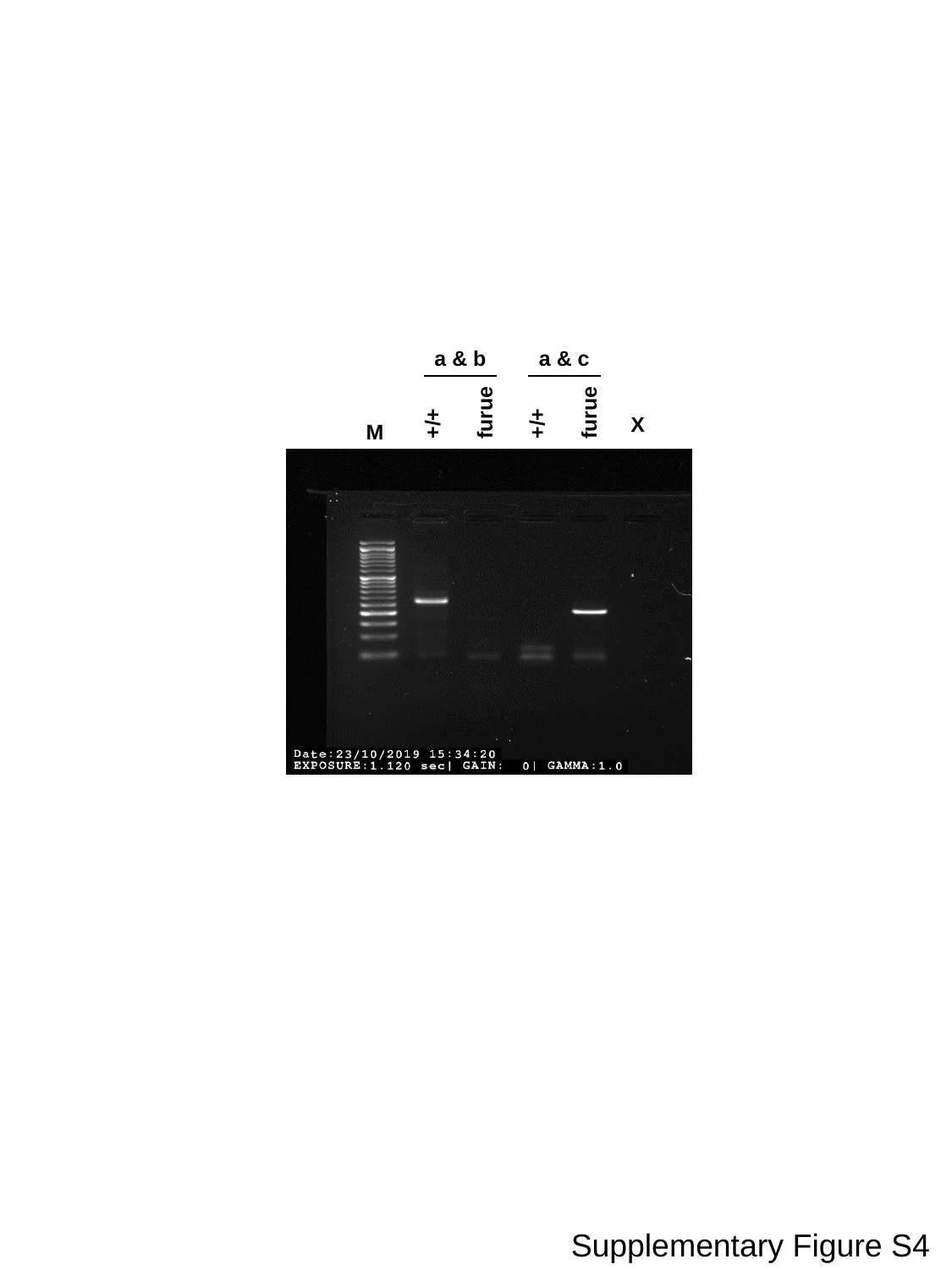

a & b
a & c
furue
furue
+/+
+/+
X
M
Supplementary Figure S4

## Slide 6
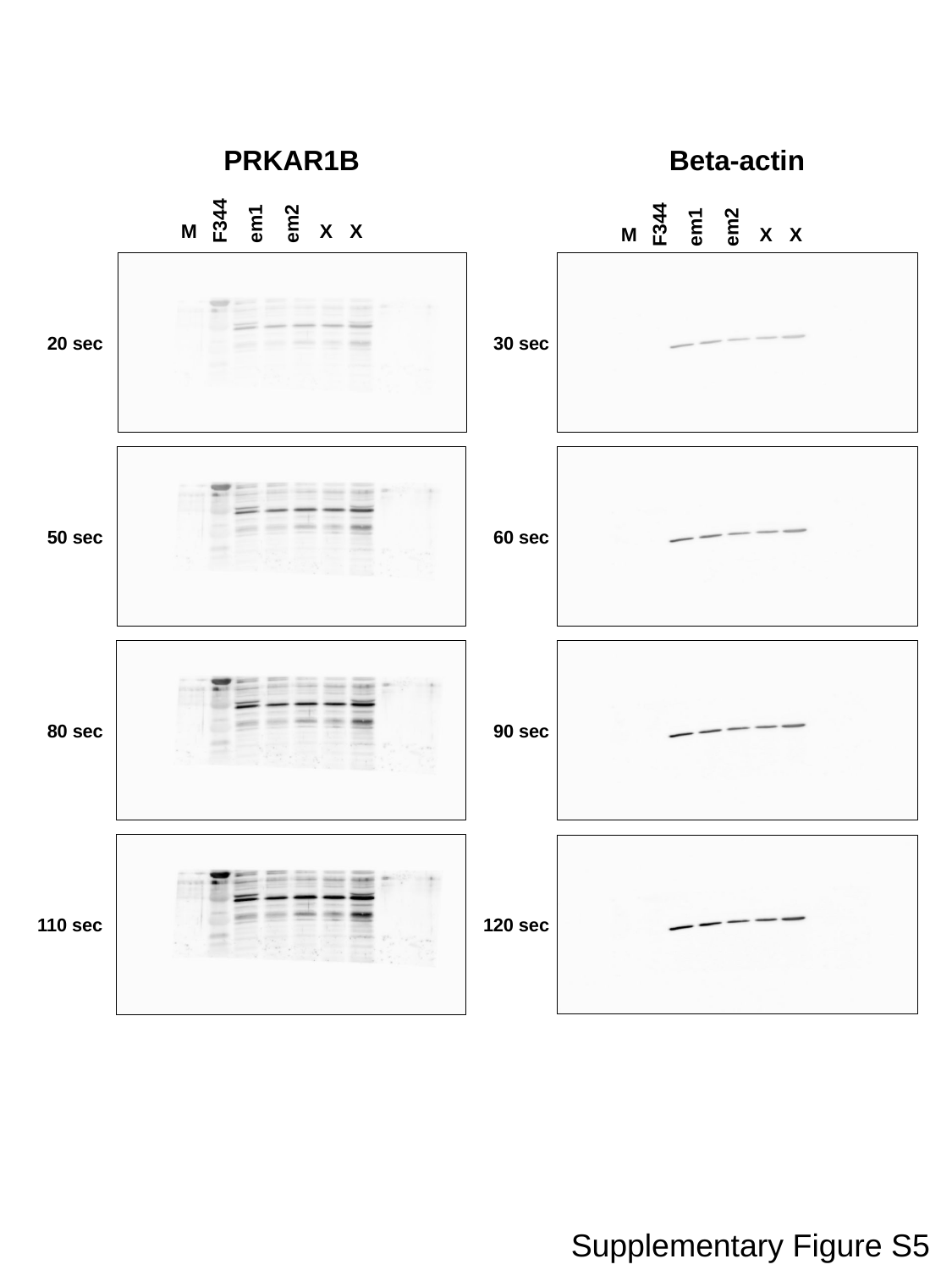

PRKAR1B
Beta-actin
F344
em1
em2
M
X
X
F344
em1
em2
M
X
X
20 sec
30 sec
50 sec
60 sec
80 sec
90 sec
110 sec
120 sec
Supplementary Figure S5
